# Social Determinants and Access to Induced Abortion in Burkina Faso: From Two Case Studies

**DOI:** 10.1155/2014/402456

**Published:** 2014-03-17

**Authors:** Ramatou Ouédraogo, Johanne Sundby

**Affiliations:** ^1^Université Bordeaux Segalen, UMR 5115 (Les Afriques dans le monde), Sciences Po Bordeaux, 11 allée Ausone, F 33607 Pessac Cedex, France; ^2^Section for International Health, University of Oslo, Post Box 1130 Blindern, N 0317 Oslo, Norway

## Abstract

Unsafe abortion constitutes a major public health problem in Burkina Faso and concerns mainly young women. The legal restriction and social stigma make abortions most often clandestine and risky for women who decide to terminate a pregnancy. However, the exposure to the risk of unsafe induced abortion is not the same for all the women who faced unwanted pregnancy and decide to have an abortion. Drawn from a qualitative study on the issue of abortion in Ouagadougou, Burkina Faso's capital, the contrasting cases of two young women who had abortion allow us to show how the women's personal resources (such as the school level, financial resources, the compliance to social norms, the social network, etc.) may determine the degree of vulnerability of women, the delay to have an abortion, the type of care they are likely to benefit from, and the cost they have to face. This study concludes that the poorest always pay more (cost and consequences), take longer to have an abortion, and have more exposure to the risk of unsafe abortion.

## 1. Introduction

The issues of inequalities between human beings have always been a topic of interest. Inequalities are perceived as at the foundation of the stratification of societies [1]. The duality wealthy/underprivileged or rich/poor seems to be part of the operating mode of human relationships, and different indicators of measures such as the Human Development Index (HDI) initiated by United Nations development Programme (UNDP) aim to identify and address this contrast. From the macrolevel (from one region of the world to another, or from one country to another) to the microlevel (a community within the same country or from an individual to another) these inequalities can be found in various fields including health. Social inequalities in health, defined by the General Inspectorate for Social Affairs of France as “*any relationship between health and the belonging to a particular social group*” [2], stipulate the existence of a link between the state of health of an individual and his social position/status. These inequalities in the health field are not just biological but also and above all are socially constructed [3].

Almost all over the world, there are demonstrable social gradients in health [4]. And the associations are invariably one-directional, namely that the poorer you are or the lower you are on the social hierarchy, the worse your health is. This is especially true for solid life and death indicators like infant mortality rate, life expectancy, under-five mortality, and likewise. People living in poor countries, at an aggregate level, have shorter life expectancy than more well-off. At the aggregate level, we also know some of the causes of ill health: improper nutrition, poor access and utilization of preventive interventions (immunization coverage), poor housing, more exposure to infectious agents, and so forth. Disparities in health based on socioeconomic status and the nature of social relationships have been demonstrated in different studies in the social and medical sciences [5–8]. These studies reveal that disparities in health are driven by a complex set of causal processes including largely social determinants. Therefore, social factors (social network, level of education, the status in the social hierarchy, and economic status, etc.) would make people more vulnerable and being at risk of diseases than others. Risk factors could be the number of cigarettes smoked, inability to buy a health insurance, poor knowledge of factors that contribute to exposure, or simply poor ability to own assets. It could also be risky behaviors like unsafe sexual intercourse or eating fat-/carbohydrate-rich foods. These risk factors affect the probability that you get unhealthy or do not get health assistance when you have a disease. To understand how social positioning, poverty, low level of education, and similar things can bring individuals being more at risk (e.g., of disease or death), we need a better close-up understanding about the pathways/mechanisms of social factors that affect health: the limitations and lack of choices people have in concrete health-hazard situations at the microlevel. In order to illustrate how this may be enacted and how different social positions may end up in vastly different endpoints from the same risk position, even in a country that is among the poorest in the world, such as Burkina Faso, we have chosen to look at a common but difficult situation for young women, namely, induced termination of pregnancy.

Induced abortion is a practice that has been used since generations as a method of fertility regulation [9]. It is practiced in all countries regardless of the level of development, the strength of family planning programs, or the legal policies that regulate it. However, factors such as legislation concerning abortion, moral convictions, poverty, and access to abortion services contribute to make some abortions “safe” and other “unsafe”. Unsafe abortion is defined by the World Health Organization [10] as any procedure to terminate an unintended pregnancy done either by people lacking the necessary skills or in an environment that does not conform to minimum medical standards or both. This category of abortion was even called “silent scourge” because of the high number of deaths attributable to it and its economic and social consequences [11]. In Burkina Faso, the legal restriction and social stigma make abortions most of the time illegal and clandestine practices which may lead to several complications and deaths [12]. But the exposure to this risk of unsafe clandestine induced abortion is not the same for all young women who intend to have an abortion.

Studies (especially quantitative) have shown that women in poor countries with low socioeconomic resources are more likely exposed to unsafe induced abortion compared to women with high socioeconomic resources in developing countries or women living in countries where there is a liberalized access to abortion [12–16].

Thonneau [17] stated that “*the risk of death from unsafe abortion is 1 per 150 in Africa, for example, while it is only 1 per 150,000 in United States and Europe.*” This situation is due to restrictive laws and lack of financial resources to pay for safe abortion.

We propose, in this contribution, further reflection on the determinants of inequalities in access to clandestine induced abortion practices in a context of legal restrictions, stigmatization, and poverty such as in Burkina Faso. By using a thick ethnography of two contrasting trajectories of women who had abortion, we will show how economic resources and also determinants such as social networks, the compliance to social norms, or the fact of being educated or not can induce a differential exposure to a same risk of clandestine abortion. This thick description could contribute to make a visible picture of “the imponderable and the invisible” (an expression used by Fassin [18]) in field of inequalities in access to induced abortion services. Indeed, qualitative research, according to Fassin [18], when applied to the field of social inequalities, through its tools such as case studies or observation, can “*reveal elements undoubtedly useful to the study of inequality, but which do not nevertheless let themselves hardly seize into a questionnaire's grid, so sophisticated it is. This elusive part, or at least un-grasped part, in statistical surveys can be analyzed within two categories: the part which does not lend itself to measurement and which may be designated as imponderable (or difficult to estimate); and the part which a priori goes unnoticed and can be considered as invisible (or partially occulted). Qualitative techniques may therefore have the dual function of identifying the imponderable and reveal the invisible.*” Within a context where reducing maternal mortality is one of the Millennium Development Goals to which the Burkina Faso's government subscribed, we assume that a thick description of these mechanisms could help to better understand the vulnerability of young women to unsafe induced abortion and provide precious guidance to improve interventions to deal with.

## 2. Materials and Methods

The aim of this paper is to demonstrate, throughout two cases studies (T and A cases), how social determinants such as personal resources (social and economic) may determine the type of clandestine abortion women are likely to access (safe or unsafe) and the time taken from knowing that a woman is pregnant to the situation of effective abortion within a country where the access to induced abortion is restricted.

The cases of T and A (both pseudonyms) are drawn from a qualitative study conducted in 2011-12 in Ouagadougou (Burkina Faso's capital). This qualitative study is part of a larger project of research focused on the issue of access to postabortion care in Burkina Faso and funded by The Research Council of Norway. Its objective was to identify and analyze the plurality of actors, norms, and practices around abortions and postabortion care, and throughout it, the process of decision making to have an abortion.

The illegality of most abortions and its clandestine nature imply difficulties in identifying women who had an abortion. Therefore, we chose to go through the entry of an institutionalized health service, postabortion care, to establish contact with women. A long immersion in three health facilities allowed us to observe the interactions between health workers and women during postabortion care performing and to identify 45 women (from 17 to 36 years old) who have been treated for incomplete induced abortion or complications of induced abortion and to conduct interviews with them. This paper is based on in-depth interviews using semistructural interview guide and informal discussions with two contrasting cases out of this sample (contrast in terms of level of education, compliance to norms, economic status, and their itineraries to perform their abortions). The consent of the women (formal or oral consent) has been asked before undertaking the interviews. The interviews have been undertaken at the health facilities, at their homes, or at their place of work, and occasionally at the interviewer home.

## 3. Results

### 3.1. First Case: Aline Fight for Timely End Safe Abortion

A, the first young woman, is a 26-year-old unmarried woman. Her father is a manager of an NGO and her mother is a housewife, and she is their only. She is a law student and is hired as a cashier (part-time) in a transport company with 150.000 fr (305$US) (1$US = 491.669 FCFA) as monthly salary. She has also the financial support of her parents and her boyfriend who is a 28-year-old single man.

When she accidentally got pregnant (contraceptive method failure), she decided to have an abortion because she did not feel ready to be mother. Her boyfriend, despite his desire to have a child, accepted her decision. She was about three weeks pregnant when she did the pregnancy test:* “I expected my periods during one week in vain. I looked at my breasts starting to swell. So I went to the drugstore, I took a pregnancy test, I did it at home and it was positive. I immediately talked with my boyfriend and he agreed that I remove it ‘I can't force you to have a child, you can do it, and be quite, I will assume all expenses, prescriptions.'”* Then, she decided to challenge norms and laws for safe abortion because she heard about the consequences of unsafe induced abortion.

*“I went immediately in the CSPS X (CSPS means Health and social promotion center. The CSPS is a primary health facility and represents the first level of Burkina Faso health system.), I met a nurse. He told me that they cannot perform abortion. I told him, ‘it's not you cannot do that, if there is a woman who is pregnant and her life is in danger, and you must choose between the child and the woman, I know you will choose to remove the pregnancy to save her live. I am not in this kind of situation that why you refuse to help me'. We discussed a long time on the law about abortion (…), and finally he said that his neighbor who is druggist told him about a drug that can be used to have abortion, but the prescription is mandatory to buy it. I beg him to find the name of the drug for me (…) and he finds it, Misoprostol! I went to the CMA (The CMA mean medical health center with surgical unit. It is a public health facility.), and read on the doors to find a doctor. I found one and I argued my case again. He told me ‘oh my sister, that the private practices deals, you may go there they will do it for you'. I told him that I have a name of a drug and I just want prescription to buy it. He said ok, he will help me, as I had a safer sex and became pregnant despite it, it's not my fault, and as it is just a prescription he can do it. He told me to go to a health facility when will I start bleeding. I did my pregnant test at 7am, and at 11am I already had my prescription!”.*

She bought the Misoprostol 5000 fr (amount in dollars US) from a drugstore, took it at home, and then she went to the clinic ABBEF two days afterward for a consultation where she openly said she had taken Misoprostol to have abortion: “*you see, I knew that the best way for them to give me the right treatment is to know from what I am suffering. I was not worried about what they would say or think about me, my objective was being treated to avoid complications.*” She paid there 7000 fr for an ultrasound scan, 3000 fr for the postabortion care, and 2075 fr for the prescription given by the midwife after the postabortion care. When she came back one week after at her appointment, the midwife consultation showed that all was fine.

### 3.2. The Second Case: T's Long Attempt of Abortion

T is a 25-year-old unmarried woman. She already has two children (both live with her mother in Bobo-Dioulasso, the second town of Burkina Faso). She left her parents' home to find job in Ouagadougou 10 years ago (at 15 years). She comes from a polygamous family and has only primary school level. When I met her, she was a waitress in a “maqui” (a bar) in Ouagadougou with 25.000 fr (50$US) as monthly salary. Her father died ten years ago and she has to bring financial support to her mother and sisters who live in Bobo-Dioulasso. Her boyfriend is a married man who rents out the house where she lives. When she found herself with an unwanted pregnancy, she decided to have an abortion because her priority now is to get married.*“I think I was about one month pregnant when I did the pregnant test in the CSPS (a primary health facility). I informed immediately my boyfriend that I am pregnant but I want to abort because I am not ready yet. He begged me not to abort but I refused, so I tried to do it in secret.*” She chose first to have a self-abortion by using two methods: “*As I did not know where to find an abortionist, I tried to do it by myself. I mixed bier and “nescafé” and drank it again and again, but nothing. One week after drinking this mixture, I decided to speak to one of my friends, I heard once that she had an abortion. She advises me to cook and drink the fiber inside the fruit of the calabash, and then my pregnant will come out fast. I believe in her and I did it, but I did not realize that I was digging my grave. It was poison! As when drink it, I started vomiting, pooing and all was blood. I asked my sister who was came from Bobo-Dioulasso to visit me to call quickly my boyfriend. He came about two or free hours later. I swear, I was going to die. I fainted two times, and they decided to take me to the hospital.*”

She was admitted first in a primary health facility, and there the midwives decided immediately to take her to Yalgado referral hospital. When she arrived at Yalgado, she said:* “they asked me what happened, I told them that I tried to have an abortion. My sister told me “do not tell them, are you crazy?” and I said that I do not want to die, it's necessary for them to know from what I am suffering, so I have to tell them.”* She spent one week in Yalgado during which she had done a lot of medical exams, had blood transfer, made an ultrasound, and paid for a lot of drugs (the MVA kit for example). She paid at the end about 87000 fr. After this episode, T thought that her abortion was successful.

Two months after her stay at Yalgado hospital, she realized that she was still pregnant:* “After leaving the hospital, I was thinking that my pregnancy went out, a big mistake! One day I felt dizzy and not well. I go to a CSPS the next day to know what wrong with me. There, the Doctor checked me and told me that the child is still there, that I am pregnant about three months and few days. I say ‘it's not true, it's not possible, I am still pregnant and I did not realize it?'”*

She tried, then, to find another way to have abortion. One of her colleagues told her about a private clinic where she can have an abortion:

*“When I went to these private clinics they said they can't do it because it is too late, over three month pregnant they cannot do abortion. They gave me the name of another private clinic, I went there and they gave me the same answer ‘we can't do it'. Finally I find another one where the doctor accepted to do it, but he asked me to pay for 65000 fr (132$US). I had lost my job at this moment, then I decided to return to my last job in the “maqui” and beg my boss to hire me, and he accepted. I started working and saving money. One month, two month, and I finally got 50.000 fr (104$US). I went back in the care practice and I begged the doctor to accept the 50.000 fr. He said okay but I have to pay for the drugs, about 20.000 fr. What can I do, I haven't no choice”.*

She describes this second attempt as very painful and complicated too.* “Given that the pregnancy was more than five months old, the doctor had difficulties to remove it, he tried during three days and the third day he succeeded. And that day it was heated, I did not have strength anymore, I was bleeding. He gave me a prescription and told me to go home. Fortunately everything is ok now.”*

## 4. Discussion

### 4.1. From Differential Resources

The “historical” lower social position of women than men at an aggregate level has been documented. They are not only affected by their own social position but also by the position of male, family members, and friends [19, 20]. As T, many women in poor countries, such as in Burkina Faso, have very limited access to schooling and employment and also a lower power in decision-making. In Burkina Faso, despite policies to improve the trends, girls are still less educated than boys and are also most affected by deschooling [21]. It is the same for the rate of inactivity or unemployment, particularly in the case of the youth [21]; as consequence, most of young people live in precarious conditions and depend on their relatives (economically and on the ability to decide). But, within this context, there will always be some of these women who manage to overcome these barriers, usually because they are born into a better socioeconomic situation such as A. Out of the 45 women we interviewed, only nine had a situation that could be described as relatively easy because they come from wealthy families or carry out activities that give them a substantial income to enable them to meet their needs. However, this does not prevent them from becoming pregnant in a time when they are not ready for it such as those in lower socioeconomic position. Unwanted pregnancy is very common, and a decision to terminate pregnancy is also common. But their therapeutic itineraries to get abortion and the exposition to the risk of unsafe induced abortion are not the same for all these women who faced unwanted pregnancy.

### 4.2. To a Differential Exposure to the Risk

The description of T and A search for abortionshows that their personal resources have led to differential itineraries in their search of abortion and differential exposure to the same risk factors which is the illegal induced abortion. Indeed, A's resources helped her to get her abortion in one week and she paid just 17075 fr (35$US), while T, after discovering her pregnancy about at the same gestation as A's pregnancy, had her abortion five months later after several attempts and paid about 186.000 frca (325$US).

When we refer to the description of the vulnerability made by Delor and Hubert [22] in the case of HIV infection, they have shown that in the study of vulnerability, three aspects have to be taken into account: the personal trajectory,* the interactions* in which one may be involved, and the* context in which the person lives*. In each three aspects, three other parameters have to be considered too: the exposure, the capacity, and the potentiality. For the personal trajectory, for example, the objective and subjective elements of the trajectory influencing exposure (such as lingering shame, feeling of urgency, and lost time), the objective and subjective contextual resources and restrictions (such as sociocultural capital, knowledge of the risk, prior experiences, etc.), and the objective and subjective consequences of infection (HIV) on the individual determine the level of risk.

In the case of abortion in Burkina Faso, the Penal Code punishes induced abortion in its Articles 383 to 386 and 388 to 390. The Article 383, for example, states “*shall be punished by *imprisonment of one to five years and a fine of 300,000 to 1.500.000 frCFA anyone by food, drink, drugs,* operations*, violence or any other means, provides* or* attempts to provide the abortion of a pregnant woman or supposed pregnant with she's consent or not. Whether death ensued,* the penalty is* imprisonment for ten to 20 years. The court may further order professional disqualification and/or residence prohibition for a period not exceeding five years” (from the Penal Code of Burkina Faso, P.61). Induced abortion is permitted only in cases where woman's life is at risk, fetal malformation, rape, and incest with juridical proofs according to the Article 387 of the Penal Code of Burkina Faso. Also, with 60.5% of Muslims, 23.2% of Christians (19% Catholic and 4.2% Protestant) [21], and pro-natalist social values, abortion as practice is subject to negative perceptions. Induced abortion is considered as a crime, a moral deviance which is socially and religiously rejected. According to a survey conducted by the Center for Democratic Governance in 2010 focused on Burkinabè and their values, induced abortion is the third practice deemed ineligible after homosexuality and prostitution. Then women who decide to terminate their pregnancy face the same potential social risk (risk of prosecution and rejection) and risk of morbidity (due to the obligation to resort to clandestine induced abortion). They are also faced with the same potential social risk of stigmatization and prosecution. But the personal resources will introduce differences in the vulnerability to this potential risk.

The school level, financial situation, the compliance to norms, the social networks, and so forth have a significant influence on the timing to get induced and effective abortion. As we saw in the case of A, she decided to challenge the law prohibition and the social reprobation and their consequences—risk of prosecution and risk of stigmatization and rejection by family and friends—to have a safe abortion. Her situation can be justified by her financial autonomy (which, e.g., help her not being afraid of losing financial support in case of rejection), her school level (she is a law student who has knowledge of the abortion law and how it works and she can find the necessary arguments to convince a health worker to help her), her status in her relationship with her boyfriend (which helped her to decide and convince quickly her boyfriend to accept her decision).

In contrast, T was in a quite differential situation: she was financially dependent on her boyfriend, had a lower level of schooling, and was in a strong logic of concealment of her abortion because of the consequences this may have on her social network. Indeed, women in situations of dependence (residential, economic, and decisional), and also because of the economic and social precariousness in which the pregnancy puts them (a weakening of their social relationships) [23], choose clandestine abortion in general because they want to keep their abortion secrete to avoid the risk of rejection and loss of support in a situation of dependence. And also because they do not have the resources required such as economic resources, social network while having an abortion is often a long process that requires considerable resources to find how to abort safely and timely (find the abortionist, ensuring costs, etc.) [24–26]. Adolescents and young unmarried women are generally concerned with this situation. Guillaume [13] has shown that young women often abort late, using unreliable methods because they do not have the means to access to safe methods. They tried to have self-induced abortion by “*using methods that are very harmful to the health or lives of women, as is proven by studies on abortion-related complications which show that women hospitalized for these reasons have for the most part used plants or overdoses of medication, or have inserted objects into the vagina.*” When these methods fail, they tried to find abortionist and this search can take long time because of the difficulties to find them and because of the lack of means to afford the costs [27] as in the case of T. Sometimes, the process of decision-making to terminate the pregnancy may take a long time in case of conflictual fertility intentions [28] or strategic pregnancies aimed at creating or reinforcing ties with a boyfriend to get married and escape socioeconomic precariousness [29]. Young single women in such situation tend to delay the decision with the hope that the man will accept the pregnancy as we saw in some cases of our sample. All these factors may delay the performing of abortion; while abortion is semiurgent care which requires urgent intervention because the risk of complications increases with gestation, abortion becomes impossible if it is delayed too long, and most women who have chosen to terminate their pregnancies want to do so as early as possible [30]. It should be noted that safe abortion is not always more expensive. Women with poor resources do not have the possibility to pay for safe abortion due to its high costs [31], but the addition of the costs throughout their abortion itinerary shows that they pay more at the end. Then, the poorest always pay more (cost and consequences), take longer to have an abortion, and have more exposure to the “risk” of unsafe abortion.

## References

[1] Rousseau J-J Les inégalités sociales de santé: Déterminants sociaux et modèles d’action.

[2] Moleux M, Schaetzel F, Scotton C Les inégalités sociales de santé: Déterminants sociaux et modèles d’action. http://www.ladocumentationfrancaise.fr/var/storage/rapports-publics/114000580/0000.pdf.

[3] Aïach P (2000). 5. De la mesure des inégalités: enjeux sociopolitiques et théoriques. *Recherches*.

[4] Marmot M (2005). Social determinants of health inequalities. *The Lancet*.

[5] Eboko F, Grenier-Torres C, Mestre C Inégalités et santé: des disparités récurrentes à un projet global?. *Face À Face Regards Sur Santé*.

[6] Goldman N (2001). Social inequalities in health: disentangling the underlying mechanisms. *Annals of the New York Academy of Sciences*.

[7] Lombrail P, Pascal J (2005). Inégalités sociales de santé et accès aux soins. *Les Tribunes de la santé*.

[8] Robert SA (1999). Socioeconomic position and health: the independent contribution of community socioeconomic context. *Annual Review of Sociology*.

[9] Guillaume A L’ avortement en Afrique: mode de controle des naissances et probleme de santé publique.

[10] WHO The prevention and management of unsafe abortion. *Report of a Technical Working Group*.

[11] Grimes DA (2003). Unsafe abortion: the silent scourge. *British Medical Bulletin*.

[12] Kaboré I, Bankole A, Rossier C, Sedgh G Characteristics of women who have induced abortions, type of providers used and the health consequences in Burkina Faso. http://uaps2011.princeton.edu/papers/111006.

[13] Guillaume A Abortion in Africa. A review of literature from the 1990's to the present days. http://www.ceped.org/avortement/gb/index800.html.

[14] Jones RK, Upadhyay UD, Weitz TA (2013). At what cost? payment for abortion care by U.S. women. *Womens Health Issues*.

[15] Singh S, Wulf D, Hussain R, Bankole A, Sedgh G http://www.guttmacher.org/pubs/Abortion-Worldwide.pdf.

[16] Toulemon L (1992). Maîtrise de la fécondité et appartenance sociale: contraception, grossesses accidentelles et avortements. *Population*.

[17] Thonneau PF Mortalité maternelle et avortements dans les pays en développement. http://www.itg.be/ITG/GeneralSite/InfServices/Downloads/shsop18.pdf#page=167.

[18] Fassin D (2000). 8. Qualifier les inégalités. *Recherches*.

[19] Bourdieu P (1990). La domination masculine. *Actes de la Recherche en Sciences Sociales*.

[20] Maurice G (1982). *La Production des Grands Hommes. Pouvoir et Domination Masculine Chez les Baruya de Nouvelle-Guinée*.

[21] http://ecastats.uneca.org/aicmd/Portals/0/Resultats_definitifs_RGPH_2006.pdf.

[22] Delor F, Hubert M (2000). Revisiting the concept of ‘vulnerability’. *Social Science and Medicine*.

[23] Ouattara F, Storeng K (2008). L’enchaînement de la violence familiale et conjugale. Les grossesses hors mariage et ruptures du lien social au Burkina Faso. *Bulletin de l'APAD*.

[24] House JS, Landis KR, Umberson D (1988). Social relationships and health. *Science*.

[25] Ostrach B The role of social support in overcoming obstacles to abortion access: oregon women tell their stories. http://scholarsarchive.library.oregonstate.edu/xmlui/handle/1957/16387.

[26] Rossier C (2007). Abortion: an open secret? Abortion and social network involvement in Burkina Faso. *Reproductive Health Matters*.

[27] Singh S, Wulf D, Hussain R, Bankole A, Sedgh G http://www.escr-net.org/node/364823.

[28] Rossier C, Sawadogo N, Soubeiga A (2013). Sexualités prénuptiales, rapports de genre et grossesses non prévues à Ouagadougou. *Population*.

[29] Ouedraogo R, Ouattara F (2013). *Vulnérabilité séquentielle des jeunes femmes face à l’avortement: incidence des normes sociales et juridiques à Ouagadougou (Burkina Faso)*.

[30] Henshaw SK (1995). Factors hindering access to abortion services. *Family Planning Perspectives*.

[31] Rossier C, Guiella G, Ouédraogo A, Thiéba B (2006). Estimating clandestine abortion with the confidants method—results from Ouagadougou, Burkina Faso. *Social Science and Medicine*.

